# Deciphering the heterogeneity of myeloid cells during neuroinflammation in the single‐cell era

**DOI:** 10.1111/bpa.12910

**Published:** 2020-11-06

**Authors:** Katharina Borst, Marco Prinz

**Affiliations:** ^1^ Institute of Neuropathology Medical Faculty University of Freiburg Freiburg Germany; ^2^ Center for Basics in NeuroModulation (NeuroModulBasics) Faculty of Medicine University of Freiburg Freiburg Germany; ^3^ Signalling Research Centres BIOSS and CIBSS University of Freiburg Freiburg Germany

**Keywords:** MS, myeloid cells, neuroinflammation, sc‐RNA‐Seq

## Abstract

Multiple sclerosis (MS) is a disabling neuroinflammatory disease, which is little understood and lacks a sufficient therapeutic regimen. Myeloid cells have repeatedly shown to play a pivotal role in the disease progression. During homeostasis, only the CNS‐resident microglia and CNS‐associated macrophages are present in the CNS. Neuroinflammation causes peripheral immune cells to infiltrate the CNS contributing to disease progression and neurological sequelae. The differential involvement of the diverse peripheral and resident myeloid cell subsets to the disease pathogenesis and outcome are highly debated and difficult to assess. However, novel technological advances (new mouse models, single‐cell RNA‐Sequencing, and CYTOF) have improved the depth of immune profiling, which allows the characterization of distinct myeloid subsets. This review provides an overview of current knowledge on the phenotypes and roles of these different myeloid subsets in neuroinflammatory disease and their therapeutic relevance.

## Graphical abstract

This review provides an overview of current knowledge on the phenotypes and roles of different myeloid subsets in neuroinflammatory disease and their therapeutic relevance.

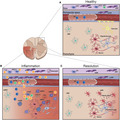

## Introduction

The autoimmune disease, multiple sclerosis (MS), is a chronic inflammatory disease of the central nervous system (CNS). The disease is characterized by demyelinated plaques in the white matter (WM) and spinal cord (SC), as well as neurodegeneration, and is the leading cause of nontraumatic neurological disability in young adults (60). It presents clinically as three different subtypes. Most patients develop relapse‐remitting MS (RR‐MS), for which disease‐modifying drugs (DMF) are available. However, about 15% of patients present with primary progressive MS (PP‐MS) and 80% of untreated RR‐MS cases develop into secondary progressive MS (SP‐MS), for which specific medication is scarce or unavailable (150). The underlying reasons for the disease induction are multifactorial, the treatment is difficult, and until today, there is no cure available. A striking feature of the disease progression is the infiltration of peripheral immune cells contributing to the endogenous CNS immune population, which results in a heterogeneous/complex immune landscape (156). The myeloid cells are considered key players in the concomitant rise in inflammation, which leads to neurodegeneration such as persisting axonal damage.

The CNS is separated from the periphery by the blood–brain barrier (BBB) but possesses a functional lymphatic drainage system. Previously thought to be immune‐separated, the CNS is currently thought of as rather immune‐privileged. Specialized anatomical structures such as the meninges, perivascular space, and the choroid plexus (CP) not only form the CNS borders but also form interfaces, which are occupied by specialized tissue‐resident innate immune cells known as CNS‐associated macrophages (CAM) (85). These interfaces are critical for immune reactions. For instance, autoreactive T cells in the meninges contact local phagocytes and ligation of lymphatic vessels inhibited disease progression and peripheral immune cell infiltration into the CNS and finally, the removal of CNS‐draining lymph nodes ameliorated EAE (108, 169, 205). Microglia, which reside in the brain parenchyma, and CAM differentiate prenatally and develop into tissue‐resident macrophages, while other myeloid cells develop in the bone marrow (BM) and infiltrate tissues after birth, for instance upon inflammation. Depending on their development, these myeloid cell subsets show very distinct phenotypes and functions under homeostatic and disease conditions.

Activated microglia have been found in early and late MS stages and reactive microgliosis, consisting of dividing microglia, has been reported in active demyelination plaques in MS patients (98, 173, 184). Additionally, several risk loci were found in MS patients, of which 48 are highly expressed in microglia as well as some specific to dendritic cell (DC), such as ZBTB46 (50, 69, 103, 166). Studies on human MS patient tissue have also reported on high levels of infiltrating myeloid cells, however, it is challenging to dissect infiltrating from CNS‐resident myeloid cells. Upon infiltration of CNS tissue and under immune stimulation, myeloid cells display an altered morphology and lose their homeostatic signature, which during steady state, helps define their ontogeny. Several excellent reviews prior to the single‐cell era have reported on the importance of myeloid cells during MS (11, 15, 47, 54, 171). However, recent technological advancements, from single‐cell tools to the improvement of preclinical models, have provided new platform for the in‐depth profiling of myeloid subsets and to assess their functional states in diseases.

While the aging CNS shows histological hallmarks of neurodegeneration such as axonal damage and concomitant microglial expansion, features reminiscent of MS pathology, microglia activation profiles associated with neuroinflammation in MS can differ from that observed in neurodegeneration (3, 58, 134). Therefore, while common pattern can be identified across CNS pathologies, the spectrum of myeloid activation states identified in MS should not be underestimated.

In this review, we provide an overview of the range of immune state myeloid cells display in MS and how cell ontogeny dictates their function.

## Myeloid Cell Lineages

With the availability of genetically modified reporter mice, tremendous progress has been achieved in dissecting the development of myeloid cell populations. Tissue‐resident macrophages in the CNS include microglia as well as CAM, namely CP macrophages (cpMΦ), perivascular macrophages (pvMΦ), meningeal macrophages (mMΦ), and dura mater macrophages (dMΦ) (85, 154, 155). Microglia and CAM are yolk sac‐derived cells, whereas monocytes, DCs, and monocyte‐derived macrophages (moMΦ) develop in the bone marrow (BM) (45, 48, 55, 57, 83, 170, 183). Additionally to the CNS‐resident macrophages, BM‐derived DCs can be found in the border regions of the healthy CNS, while BM‐derived monocytes and moMΦ are only found in the CNS upon inflammatory insult. Development and most importantly function of these different myeloid cell subsets are distinct, and therefore, need to be analyzed separately.

### CNS‐resident myeloid cells

Microglia are the sole occupants of the CNS parenchyma during homeostasis. They are long‐lived and do not show turn over with blood monocytes, but rather self‐renew under homeostasis (2, 17, 48, 59, 67, 127, 180). They are the innate immune system of the CNS and important for the defense against pathogens, synaptic pruning, myelin homeostasis, and clearing of dead cells (9, 56, 142). They present a distinct ramified morphology and during homeostasis constantly monitor their surrounding environment (137). Resting microglia display a rather immunosuppressive phenotype, but are rapidly activated upon stimulation, which includes downregulation of core homeostatic microglia genes, such as the purinergic receptor P2Y12 (P2RY12) or transmembrane protein 119 (TMEM119), and upregulation of genes involved in phagocytosis, lipid metabolism, and antigen presentation (31, 91, 137). As other tissue macrophages, they express pattern recognition receptors and other immune receptors, phagocytose, and produce cytokines and chemokines (64, 86, 96, 196). However, microglia present a distinct disease‐associated phenotype (DAM), which can be neuroprotective or neurotoxic depending on the insult (26, 27, 35, 82). This phenotype can be influenced by intrinsic as well as extrinsic factors and can be readjusted in new environments, such as inflammation (10, 65, 101, 118).

CAM also develop in the yolk sac before birth and most subpopulations are long‐lived, however, in contrast to microglia, CAM are only found at the interfaces of the CNS and not in the parenchyma (48, 154, 183). All CAM are morphologically distinct from microglia and show a limited motility (48, 85, 167). CAM can be distinguished from microglia by the expression of the mannose receptor MRC1, the phagocytic scavenger receptor CD36, and lymphatic vessel endothelial hyaluronan receptor 1 (LYVE1) (37, 48, 134, 202).Recently several studies have analyzed the CAM compartment in a more detailed fashion and found distinct subsets of CAM in specific regions of CNS‐associated tissues. A detailed mass cytometry (CYTOF) study found distinct markers of CAM in the CNS‐interfaces, which consisted of c‐mer proto‐oncogene tyrosine kinase (MERTK), MRC1, CD64, F4/80, CD44, and CD16/32 (134). Additional subpopulations could be separated by their differential expression pattern of CD38/LYVE1, MHC class II, and CCR2, and these subpopulations were either enriched in the dura mater or in the pia mater and the perivascular space. Using single‐cell‐RNA‐Sequencing (sc‐RNA‐Seq) Jordão and colleagues reported a CAM core signature in all CAM during homeostasis, which again included MRC1, as well as platelet factor 4 (*PF4*), membrane spanning 4‐domains A7 (*MS4A7*), stabilin‐1 (*STAB1*), and carbonyl reductase 2 (*CBR2*) (74). Notably, this core signature changed upon autoimmune neuroinflammation. Complementing these sc‐RNA‐Seq data, van Hove and colleagues additionally established a high‐dimensional flow cytometry panel, which allowed the differentiation of 5–6 subsets of CAM within the specific tissues during homeostasis (185). While dMΦ and one subset of cpMΦ are originally derived from the yolk sac and later replaced by BM‐derived monocytes, this study proposed that Kolmer epiplexus cells are not a prototypical CAM but rather a specialized subset of microglia residing in the CP. It has been difficult to address the specific function of CAM so far, but a recent review has summarized the potential functions within the indicative data available (85).

### Peripheral myeloid cells (monocyte/macrophage/DC)

Human monocytes can be separated in CD14^+^CD16^−^ so called classical, CD14^+^CD16^+^ and CD14^low^CD16^+^ nonclassical monocytes (143). In contrast, mouse monocytes are characterized by different surface markers, but the subsets display the same characteristics as their human counterparts. Classical mouse monocytes are Ly6C^hi^CX3CR1^int^CCR2^+^CD62L^+^CD43^low^, whereas nonclassical monocytes are Ly6C^low^CX3CR1^hi^CCR2^low^CD62L^−^CD43^+^ (41, 72, 76, 141). Additionally, two recent studies using sc‐RNA‐Seq implemented more subsets within the current classification (129, 186). The differentiation of monocytes occurs from hematopoietic stem cells via several steps in the BM before mature monocytes leave the BM (55). Classical circulating monocytes are only sustained in the blood for a short time before they either infiltrate tissues and differentiate into tissue‐resident macrophages/DCs or differentiate into nonclassical monocytes (165, 200).However, this is not the case in the CNS where monocytes are not present under homeostatic conditions. Upon neuroinflammation, they are able to infiltrate the inflamed CNS tissue and gain an inflammatory DC or macrophage (MΦ)‐like signature, and are therefore, considered as moDCs or moMΦ, respectively. These blood‐derived myeloid cells integrate the local tissue myeloid cell pool temporarily (3, 74).

DC development is now classified as bona fide DC, including plasmacytoid DC (pDC), classical DC1 (cDC1 (nonclassical MHC‐I cross‐presentation), cDC2 (classical MHC‐II presentation)) subsets, and as monocyte‐derived DC (moDC). Newly established markers can now differentiate cDC1 and cDC2 subsets during homeostasis, via the expression of the c‐type lectin‐like receptor CLEC9A and zinc finger and BTB domain containing 46 (ZBTB46), respectively (123, 161, 164). cDC1, cDC2, and pDC are found in the leptomeninges, dura mater, and CP in the healthy CNS (4, 53, 134, 135). Jordão and colleagues did not find pDC in the healthy CNS, probably because of under‐sampling, however, they established a core gene signature for DCs on a single‐cell level during EAE, which includes basic leucine zipper ATF‐like transcription factor 3 (*BATF3*), *CD103*, *FLT3*, *ZBTB46*, and *CLEC9A* (74).

## Experimental Models for MS

Experimental rodent models have been the primary platform to study MS with as the most commonly used MS models experimental autoimmune encephalomyelitis (EAE), toxin‐induced demyelination, including cuprizone (CPZ) or lysolecithin (LPC), and Theiler’s murine encephalomyelitis virus model (TMEV). One has to keep in mind that all these models mimic only specific aspects of the human disease and several recent reviews have focused on the detailed description of these different rodent models and their shortcomings (99, 114). Here, we outline the most important characteristics of the most frequently used models.

EAE can be induced in different rodent strains, with different resulting clinical phenotypes, with the C57BL/6 mouse being the most common although not the most representative. Myelin oligodendrocyte glycoprotein (MOG)_35‐55_ causes monophasic EAE with incomplete recovery and it is easy to perform and reproducible in C57BL/6 mice (46). MOG_35‐55_ is injected subcutaneously to induce active EAE, whereas for induction of passive EAE, disease causing autoreactive CD4^+^ T cells are adoptively transferred into naïve mice. In these settings mice develop a chronic form of EAE and most studies use primarily female animals, as male mice are more resistant to the development of EAE. In contrast, myelin proteolipid protein (PLP)_139‐151_ in SJL mice induces a relapse‐remitting disease phenotype. All the EAE models represent an inflammatory disease setting with infiltration of peripheral immune cells, thereby allowing to understand the complex interactions between CNS‐resident cells with the peripheral immune system. EAE induction leads to focal lesions of demyelination and axonal degeneration, which leads to stepwise paralysis of the animals, which can be monitored by a scoring system. Importantly, MS is dominated by an inflammatory reaction of CD8^+^ T cells and B cells, which do not play a major role in EAE (111).

In order to specifically study the process of demyelination without ongoing systemic inflammation, many studies rely on the CPZ model. CPZ is a copper chelator, which induces selective damage to oligodendrocyte progenitor cells (OPC) and leads to chronically activated microglia (174). In contrast to EAE, females are more resistant to CPZ than males. Demyelination is induced by oral administration via CPZ‐containing chow, which makes it a noninvasive form of treatment. In contrast to EAE mice, CPZ‐induced demyelination does not display any clinical signs of pathology. On the contrary, the time course of demyelination and the location of the lesions (corpus callosum) are highly reproducible. Another widely used model is LPC injection into the SC or corpus callosum, which induces location‐specific demyelination with subsequent remyelination (99). The toxin directly damages lipid‐membrane‐rich myelin sheath. The model is highly reproducible in inducing focal lesions at defined locations, but the procedure is more invasive than the CPZ treatment.

A third established model is the Theiler’s murine encephalomyelitis virus (TMEV) infection, which leads to spontaneous development of inflammatory demyelination in the SC (42). The pathogenesis is complex, since active virus infection already impacts the immune system independently of MS‐related stimulation, and as in the previous models, this system is inconsistent across mouse strains.

An additional model system which more closely represents pattern of MS disease is the common marmoset model (178). Of course, experiments conducted on primates are often not feasible. Therefore, whereas all these models display shortcomings, they all allow the analysis of specific aspects of MS pathology. Overall, a combination of data from all the models would ensure a broad insight into the disease mechanisms.

## Myeloid Cell‐Specific Mouse Models

As mentioned above, recent studies have shown that the core gene and surface marker expression of different myeloid cell populations are not stable upon inflammation. It is therefore crucial to use the most appropriate mouse model, possibly tamoxifen‐inducible Cre (Cre‐ERT2) lines, in order to answer the question of interest. Four main questions arise when choosing the right mouse model: (i) which cell type should be targeted, (ii) at what time point, (iii) are there any unspecific recombination events in other cell types, and finally, (iv) what is the targeting efficiency in the preferred cell type?

A well‐established and extremely successful mouse model to label CNS‐resident macrophages has been developed and established by Jung and our group (49, 200). The fractalkine receptor‐driven Cre‐ERT2‐induced mice (CX3CR1‐Cre‐ERT2) show very high and specific labeling in most floxed mouse lines. However, one needs to keep in mind, that this model also labels CAM, some tissue macrophages in the periphery as well as monocytes until they are replaced by new ones from the BM (4 weeks) (48). Because of these shortcomings, an array of new mouse lines have been established in order to target microglia in the CNS by using seemingly microglia‐specific Cre‐drivers (SALL1, P2RY12, TMEM119, and HEXB) (20, 77, 115, 120). The targeting specificity of these mouse lines has been nicely compiled in two recent reviews (8, 131). Future studies, specifically in inflammatory settings, will show their value and establish these mouse models as a replacement of the CX3CR1‐driven mouse model.

While mouse models which potentially target CAM but not microglia have been developed, most of them show high off‐target recombination in cells other than microglia. These include PF4‐Cre, MRC1‐Cre‐ERT2, LYVE1‐Cre, and CD169‐Cre mice (80, 120, 136, 147, 149, 181). To perform detailed fate‐mapping studies Cre‐ERT2 mice are of advantage for pulse labeling, whereas for gene deletion studies a preferably cell type‐specific marker with little off‐target deletion is needed.

Also mouse models, which target myeloid cells of the periphery, such as CD11c‐Cre, CCR2‐CreERT2, and LYZ2‐CreERT2, are often not specific enough or the targeting efficiency is suboptimal (122, 172). Therefore, it is necessary for functional studies to check both sides of the immune system in order to eliminate the potential impact of either peripheral or resident myeloid cells. Lately, new mouse models have been created, which target myeloid cells in the periphery more specifically and might help to distinguish the role of infiltrating myeloid cells from CNS‐resident myeloid cells, namely MS4A3‐Cre and ZBTB46‐Cre (104, 107). Additionally, a new mouse model targeting CXCR4 can be used to fate map peripheral myeloid cells, however, the mouse model targets all hematopoietic cells, and is therefore, not suitable for gene deletion studies (193, 198).

## The Role of Myeloid Cells in Neuroinflammation

Core signature markers have been established for most myeloid cells, which allow specific tracking of these cells under homeostatic conditions. However, in diseases, high‐dimensional cytometry (FACS and CYTOF) and RNA‐Seq in combination with algorithm‐guided analyses have shown that the myeloid landscape within the CNS undergoes phenotypic changes, which make it rather difficult to differentiate specific cell subsets via their established core markers (4, 15, 48, 54, 116, 117, 134).

For example, microglia and CAMs lose surface marker expression of their core markers (P2RY12, TMEM119 for microglia, MRC1 for CAMs) but upregulate surface markers, which are typical for DCs (CD11c) or for monocytes (LY6C) (9, 10, 18, 29, 61, 74, 113). Although TMEM119 is a microglia‐specific surface marker in healthy mice and humans, in MS lesions microglia presumably lose TMEM119 expression, which makes them indistinguishable from infiltrating myeloid cells (163, 204). On the bright side, CD49D has been shown to be helpful for the distinction of moMΦ, and CD44 is considered a specific surface marker for peripheral immune cells in homeostasis and inflammation allowing a better differentiation of infiltrating cells from resident cells in experimental studies (3, 43, 74, 134). Future studies which attempt to analyze specific myeloid cell subsets during CNS inflammation need to take advantage of cell‐specific reporter mouse lines, as lineage‐tracing is not possible in human samples.

### Microglia in neuroinflammation

In the past, microglia were shown to have distinct tasks during neuroinflammation. They are activated before the infiltration of peripheral T cells and monocytes occur, but are rather inactive during the early phase of EAE (49, 62, 153). However, as the disease progresses they are functionally important for remyelination by clearing myelin debris via phagocytosis (140, 188, 197). Some of these conclusions were drawn from experiments with BM chimeric mice, which makes interpretation of the results difficult since irradiation induces an artificial influx of circulating cells, a strong cytokine/chemokine response in the CNS, and a transient leakage of the BBB (84, 128). However, genetic depletion of microglia or deletion of genes in CNS macrophages obviously attenuated EAE (49, 62, 138).

**Figure 1:**
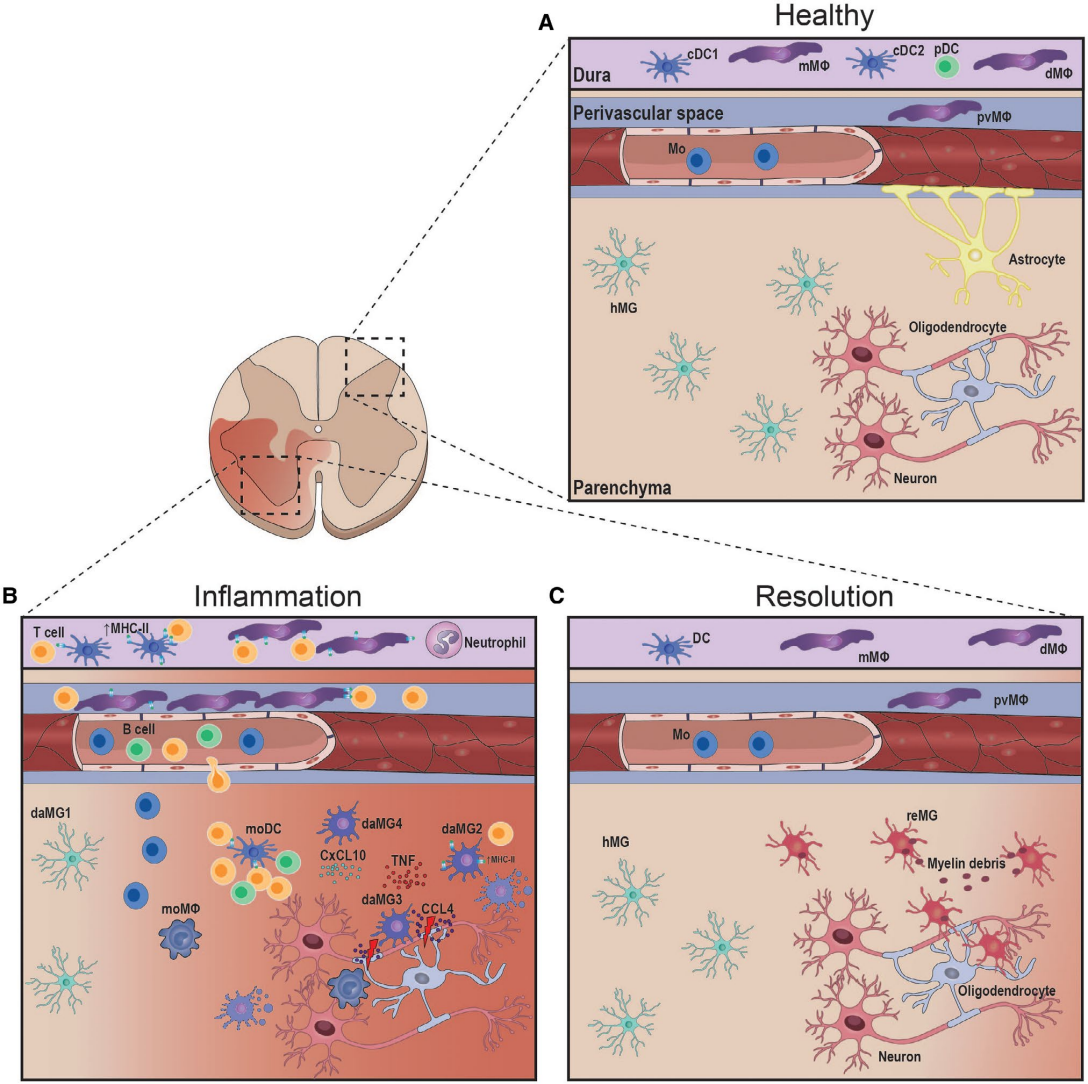
*The myeloid cell landscape in the CNS changes upon neuroinflammation*.Schematic depiction of the spinal cord during homeostasis, inflammation, and resolution. During homeostasis microglia (hMG) occupy the parenchyma of the central nervous system (CNS), whereas meningeal macrophages (mMΦ), dura macrophages (dMΦ), perivascular macrophages (pvMΦ) (CAM), and classical DC (cDC1, cDC2, and pDC) are found in the CNS‐associated tissues. Upon inflammation, microglia get activated, upregulate MHC‐II (daMG2), and produce chemokines (daMG3 and daMG4), specifically in the lesion areas. Peripheral immune cells (T cells, B cells, monocytes (Mo), and DC infiltrate into the CNS tissue. Mo differentiate into mo‐derived DC (moDC) and macrophages (moMΦ) and exacerbate neuronal damage. CAM proliferate, upregulate MHC‐II, and interact with infiltrating T cells. During inflammation microglia undergo apoptosis and are replaced by resolution supportive microglia (reMG), which phagocytose myelin debris and support oligodendrocyte precursors in remyelination processes.

Recently, several studies have established a comprehensive map of the myeloid cell landscape during neuroinflammation using high‐dimensional FACS/CYTOF and sc‐RNA‐Seq as a basis for more detailed analysis of specific myeloid cell function (Figure 1). The first study used the CX3CR1‐CreERT2‐YFP fate‐mapping mouse model in an EAE model and performed CYTOF analysis (3). The authors reported three distinct resident myeloid cell (microglia and CAM) populations, namely a quiescent CD317^+^MHC‐II^–^CD39^low^CD86^–^ population, a population characterized by CD317^+^MHC‐II^–^CD39^hi^CD86^+^ expression, and a population only present during disease, which expressed CD317^+^MHC‐II^+^CD39^hi^CD86^+^ as well as CD11c^+^. Importantly, this signature was distinct from a core signature previously found in neurodegenerative disease models (82, 94). A second study used the SALL1 reporter mouse model and also employed CYTOF for the analysis of microglia during EAE (134). The authors found that microglia downregulated homeostatic core markers and upregulated activation markers, such as CD44, CD86, CD274, and CD11c. Again, the authors describe a phenotypic signature distinct from the core signature of neurodegenerative‐associated microglia and reported a more general activation of the microglia population when compared to microglia during neurodegeneration. Building on the findings of these two studies, a more recent study also used the CX3CR1‐CreERT2 fate‐mapping tool for sc‐RNA‐Seq analysis of different disease states during EAE (74). It reported a microglia core signature, which includes previously described microglia genes. Upon disease onset, only olfactomedin‐like 3 (*OLFML3*) and secreted protein acidic and cysteine rich (*SPARC*) were stably expressed in microglia, whereas the other core genes were downregulated. Additionally, microglia upregulated disease‐associated genes (*LY86 (MD‐1), CCL2, CXCL10, MKI67, and CCL4)* and could be separated in four distinct disease‐associated clusters. Highly proliferative and chemokine producing microglia clusters were histologically detectable in the EAE‐induced lesions, which allowed a detailed description of functionally distinct microglia subsets in a spatial manner. These data were reinforced by a sc‐RNA‐Seq study, which used LPC‐induced demyelination as a model (58). In accordance with the previous studies, the authors report downregulation of homeostatic microglia core genes, upregulation of distinct disease‐associated genes (*APOE, SPP1, and LPL*), and a distinct core gene signature when compared with neurodegenerative microglia. Disease‐associated clusters included microglia subsets which were specifically proliferative (*BIRC5*) or chemokine producing (*CXCL10 and CCL4*). Of note, in active demyelinated lesions of human MS samples, CCL4^+^ TMEM119^+^ clusters of microglia were found, translating the mouse data into a human setting (58). CCL4 is known to attract immune cells and could therefore play a vital role in disease progression (36). Masuda and colleagues used sc‐RNA‐Seq in combination with high‐resolution histological validation and found three microglia clusters specific for CPZ‐induced demyelination, which were once more distinct from neurodegenerative and healthy samples (116). This study also confirmed that homeostatic core genes, such as TMEM119 are downregulated upon inflammation. Of note, these results were recapitulated in human MS samples and cluster analysis of human samples showed a correlation with the clusters found in CPZ‐induced mice.

This detailed description of microglia states during disease will allow the analysis of the function of these cells in more detail in the future. All the above studies found clusters of microglia, which specifically express cytokines and chemokines, such as tumor necrosis factor (TNF), CCL4, and CXCL10. TNF is known to have neurotoxic functions, CCL4 recruits peripheral immune cells, and upon EAE induction microglia upregulate STING expression and produce IFN‐I and CXCL10 (119). Several studies have focused on the role of TNF signaling in the CNS and autoimmune diseases (52, 133). It was previously shown that inhibition of soluble brain TNF promotes remyelination by increasing myelin phagocytosis by Iba1^+^ cells by systemic administration of an inhibitor (79). However, it has been proposed that TNF elicits opposing function on microglia and macrophages (39). A more detailed analysis of the differential functions of cytokines and chemokines will increase our understanding of differential myeloid cell function and potential drug targeting options.

For many years, the supposed microglial capacity to present antigens via MHC class II was a highly debated topic. Homeostatic microglia do not express MHC class II, however, upon inflammation, microglia upregulate MHC class II‐related genes and markers. Addressing the role of AG presentation specifically in CNS‐resident myeloid cells has been difficult, but several recent studies took the challenge and reported similar results. Wolf and colleagues showed that antigen‐presentation of microglia *in vivo* does not activate T cells in the CPZ model (195). Shortly after, two independent studies confirmed that microglia do not participate as antigen presenting cells (APCs) in EAE, but that this function is rather performed by infiltrating myeloid cells or DC (74, 135).

After antigen presentation and induction of disease, the next step is the resolution of tissue damage and several studies have focused on the role of microglia during remyelination. The triggering receptor expressed on myeloid cells 2 (TREM2)/apolipoprotein E (APOE) pathway has been in the focus of microglia analysis since it was shown that this pathway is important during Alzheimer’s disease progression as well as during EAE/MS (82, 94). Therefore, several studies analyzed the role of this pathway in microglia during remyelination. For instance, in CPZ‐treated TREM2^−/−^ animals, microglia were not activated by myelin lipids, and therefore, did not expand or respond to demyelination, which led to impaired remyelination (22, 152). A recent study, however, showed that microglia of TREM2^−/−^ animals are still activated and take up myelin, but fail to upregulate lipid metabolism genes and accumulate cholesteryl ester derived from myelin cholesterol, which led to neuronal damage (139). The same study showed that this phenotype could be rescued by inhibition of cholesteryl ester formation or increased cholesteryl ester transport from the cell lumen. Another study supports this notion, since APOE^−/−^ animals showed decreased remyelination capacity because of cholesterol crystal formation in myeloid cells, which in turn induced inflammasome activation (23).

Another important function of microglia in this regard is their interaction with oligodendrocytes. It has been shown just recently that, microglia are supportive of oligodendrocytes even in the healthy brain where they promote both oligodendrogenesis and homeostasis (56, 194). In contrast, during EAE activated microglia damage oligodendrocytes and neurons (14, 19, 68, 146, 187) and after the initial demyelination, oligodendrocyte progenitors cells (OPCs) are recruited and influenced by microglia. A study in 2012 used bulk‐RNA‐Seq to analyze the microglia phenotype in the CPZ model (140). This study reported a remyelination‐supportive microglia phenotype including clusters specific for phagocytosis of apoptotic cells and myelin debris (*LRP1, CALR, CD14* and *ITGB2, ITGAM, and LGALS3*), salvage of myelin constituents (*HMGCS2, LPL, and APOE*), recruitment of OPC and trophic support for the remyelinating oligodendrocytes (*CXCL10, CXCL13, IGF1, TGFB, PDGFA,* and *PDGFB*), and tissue remodeling (*MMP12 and MMP14*). Of note, during the remyelination phase after LPC treatment also Hammond and colleagues reported a microglia state expressing *APOE, SPP1,* and *LPL* (58). Additionally, a hallmark study showed in 2013 that during the early phase of myelin repair (OPC recruitment) microglia display a pro‐inflammatory phenotype (iNOS^+^, TNF^+^, and CD16^−^CD32^+^), whereas in the remyelination phase microglia show a regenerative phenotype (ARG1^+^, IGF1^+^, and MRC1^+^) (130). Recently, it was reported that upon demyelination microglia undergo necroptosis before the CNS is repopulated by anti‐inflammatory local microglia proliferation (105). If necroptosis is blocked, regeneration is concomitantly decreased. However, in human MS samples the picture becomes more complex, with CD68^+^ cells undergoing necroptosis as well as PU1^+^ cells repopulating all lesion types (105).

A very recent study has analyzed the role of microglia from a different point of view, looking specifically at neurodegeneration during MS. In this study mouse and marmot models were used to show that microglia are responsible for synapse loss in MS (192). Concentrating on the role of complement factors, the authors showed that during EAE microglia phagocytose synapses in a complement‐dependent manner. Accordingly, it was previously shown that C1q and C3 co‐localize with synaptic proteins in postmortem MS brains (125).

### CNS‐associated macrophages during neuroinflammation

CAMs are localized between laminin‐positive endothelial and glial basement membranes at the barriers of the CNS, and therefore, are particularly interesting in CNS diseases with peripheral immune cell infiltration (154). CAMs express MHC class II and were therefore thought to be capable of antigen presentation (15, 51, 89, 100, 185). Indeed, imaging of pvMΦ/mMΦ revealed physical interactions with pathogenic T cells during EAE (6). It was further reported that during chronic EAE, meningeal infiltrates and inflammatory mediators decrease during the remission phase, and inflammatory foci are the best predictors of clinical relapses (159, 160). In MS as well as EAE, accumulation of immune cells within the meninges has been observed before onset of pathology (2, 16, 190). Along these lines, it has been shown, that mMΦ produce ligands, which allow T cell adhesion (169). Accordingly, blocking these ligands decreased T cell infiltration into the CNS. Furthermore, in MS lesions MHC class II is upregulated in macrophages (12). However, several studies analyzed the impact of antigen presentation of CNS macrophages by ablation of MHC class II expression on CX3CR1^+^ MΦ, including CAMs and could show that MHC class II expression on these cells is not relevant for disease induction (74, 135, 195). However, CAMs were clearly activated and proliferate locally within lesions upon EAE induction (3, 74, 134). The CYTOF study by Mrdjen and colleagues reported an upregulation of activation markers such as MHC class II, CD44, and CD11c upon EAE induction. In addition, Jordão and colleagues found that CAMs downregulate their homeostatic core gene signature, with only MS4A7 stably expressed in EAE (74, 134). Overall, gene signatures of CAMs of different locations did not differ greatly. The questions remains whether CAMs have a specific role in autoimmune neuroinflammation? This question will hopefully be answered with the help of integrated multi‐omic studies and the use of the newly established CAM‐specific mouse models.

### Dendritic cells and their role during neuroinflammation

DC are professional antigen presenting cells, which present antigens to T cells via MHC class II and co‐stimulatory molecules. During homeostasis, cDC are not present in the parenchyma of the CNS, but few do reside in the borders of the CNS, the meninges and the CP (134) DC have been shown to play a critical role during autoimmune inflammation in the CNS, since they present antigens to T cells in the secondary lymphoid organs in the periphery, induce transmigration of autoreactive T cells into the CNS, and further stimulate T cells locally in the meninges (63, 172). Antigen presentation and subsequent monocyte recruitment to the CNS by DCs alone is sufficient for disease progression (28, 53). Although CD11c is not an entirely specific marker, the majority of DCs express CD11c and depletion of CD11c^+^ cells reduces disease severity, which is accompanied by less pathogenic T cells within the CNS (44, 144). CD11c^+^ DCs in the inflamed CNS mainly present a moDC phenotype and are usually negative for the cDC2 marker ZBTB46 (28, 73). However, it was later shown that DC regulate programmed cell death protein 1 (PD‐1) expression on T cells and thereby also control of inflammation and induction of regulatory T cells (145, 199).

A recent publication demonstrated that cDCs sample and present myelin antigens in the healthy CNS allowing parenchymal T cell entry, which initiates neuroinflammation (135). These findings are supported by the studies of Jordão and Mrdjen, which demonstrated that MHC class II expression of DC but not microglia or CAMs is important for EAE development (74, 134). Unfortunately, while core markers such as ZBTB46 are downregulated upon inflammation, moDC express typical monocytes and DC markers, which makes it very difficult to separate different subsets of DC during neuroinflammation. A recent study, concentrating on CD11c^+^ cells, established bulk‐RNA‐Seq‐based clusters of classically and alternatively activated DC, demonstrated that the disease state correlated with the expression of alternatively activated markers (191).

### Monocytes/macrophages

During autoimmune neuroinflammation monocytes are critical for development of the pathology. Initially, it was shown that CCR2^−/−^ mice do not develop EAE (34, 70). Later on, additional studies showed that monocytes are continuously recruited to the inflamed CNS, and are pro‐inflammatory, cytotoxic, and pathogenic (2, 28, 74, 87, 127, 197). T cell overexpression of granulocyte‐macrophage colony‐stimulating factor (GM‐CSF) drives monocytes toward a MHC‐II^+^CD11c^+^ phenotype, which leads to CNS infiltration and tissue damage via production of inflammatory mediators such as reactive oxygen species (ROS) (175). Recently, a large‐scale study of MS patients described a GM‐CSF producing subset of CD4^+^ T cells as a potential biomarker for MS (38).

Once in the CNS, monocytes differentiate into moMΦ/moDC, which express MHC class II, interleukin 1β, and TNF (127, 197). Furthermore, they produce proteolytic enzymes and actively phagocytose, thereby increasing demyelination (179, 197). While detrimental, on the one hand, their phagocytic function can be beneficial in eliminating myelin debris. The more myeloid cells are found in demyelinated areas the more severe is the phenotype of the EAE (2). A variety of recent studies have focused their attention on peripheral monocytes and their role in neuroinflammation. Aarts and colleagues found that lack of costimulatory receptor CD40 on LYZ2 expressing cells leads to reduced pathology in EAE, including less CD45^+^ infiltrates and dampened demyelination (1). The immense impact of peripheral myeloid cells for CNS health has been shown by Lund and colleagues. Upon microglia depletion via diphtheria toxin administration in CX3CR1‐Cre‐ERT2^‐/+^R26DTA^‐^^/+^ mice, peripheral myeloid cells are recruited to the CNS. Mice devoid of transforming growth factor‐β1 (TGFβ1) signaling in myeloid cells (LYZ2‐Cre) developed progressive and fatal demyelinating motor disease (110). However, a similar but delayed phenotype is observed when depleting TGFβ1 signaling in CNS‐resident macrophages (CX3CR1‐CreERT2). A new CYTOF study revealed that during EAE five different monocyte populations are found in the diseased CNS (3). Monocytes depict increased expression of phosphorylated STAT3 when compared to CNS‐resident myeloid cells. Of note, mice devoid of STAT3 signaling in LYZ2 expressing cells do not develop EAE, whereas mice lacking STAT3 signaling in CX3CR1 expressing cells develop EAE compared to control animals (109). Ajami and colleagues also found that CD49D and CD49E are exclusively expressed on infiltrating myeloid cells and treatment with anti‐CD49E antibody attenuates EAE severity. A further novel study has shown that not Ly6C^hi^ monocytes but monocyte precursors from the BM might directly impact neuroinflammation (43). Using sc‐RNA‐Seq, the authors identified a subset of CXCL10‐producing monocytes in the CNS during EAE, which drives EAE development. Interestingly, several other studies showed that activated microglia are also capable of expressing high levels of CXCL10 during EAE (74, 121). CXCL10 induces the recruitment of immune cells, and therefore, independent of the cell type, could be an interesting therapeutic target. Furthermore, this and other studies found Arginase 1^+^ (ARG1^+^) and Nitric oxide synthase 2^+^ (NOS2^+^) cells in the inflamed CNS, but interpreted their presence and potential function differently (43, 74, 106). Locatelli and colleagues provided insights into the longitudinal development of moMΦ in EAE. Upon CNS entry, infiltrating monocytes first display a pro‐inflammatory (iNOS^+^), then, an intermediate and later an anti‐inflammatory (ARG1^+^) phenotype (106). This shift is induced by local mediators of astrocytes but not microglia, and accompanied by metabolic changes in the different cell subsets (102). Of note, phagocytes in the meninges or perivascular space do not show iNOS^+^ expression and could represent unresponsive CAM.

## Myeloid Cells in MS

In contrast to murine microglia, microglia of presumably non‐diseased humans are already slightly activated at both histological and transcriptional level (40, 116, 162, 204). In general, microglia activity is strongly dependent on the status of the disease and their specific anatomical location (21, 146). Although human microglia express specific markers during homeostasis, such as P2RY12 and TMEM119 (10, 126), upon activation and inflammation human microglia lose the expression of these typical core markers (10, 116, 126, 204). Zrzavy and colleagues performed a detailed description of myeloid cells in different MS stages of lesion using immuno‐labeling of specific markers combined with morphological analysis and a microarray of microdissections. Their data indicated that in active MS lesions the majority of phagocytic cells are microglia, while in later stages additional peripheral myeloid cells are recruited (204). Another study focused on the regulation of TMEM119 and P2RY12 in different lesions in MS patients (189) found distinct differences between WM lesions and GM lesions, concluding that these differences might be because of the microenvironment. This observation was shared by a study which analyzed non‐lesion sites in the WM and GM of healthy individuals and MS patients in order to find indications of lesion formation (151). This study indicated that microglia change their expression pattern even before lesion formation. GM microglia of MS patients displayed increased expression of genes associated with glycolysis and iron homeostasis, whereas WM microglia showed increased lipid metabolism, similar to what is reported from active lesion microglia. A recent sc‐RNA‐Seq study analyzed whole brain tissue of MS patients and described a cluster of MS‐specific microglia‐enriched in activation markers, which co‐localized to chronic active boundaries of subcortical MS lesions (168). However, because of the nature of the study, microglia were underrepresented. An additional study performed sc‐RNA‐Seq and histology specifically on microglia of MS patients and reported disease‐specific clusters, with downregulated homeostatic core genes (116). Focusing on MS patients with a progressive disease course, a new study performed CYTOF and bulk‐RNA‐Seq in active lesions of patients (13). In contrast to results from RRMS patients, the authors found only few moMΦ even in active lesions of PMS, although these cells displayed, as described before, a foamy phenotype. Additionally, most microglia kept their homeostatic phenotype with only few changes in a TNF^hi^ subcluster and an increased phagocytic phenotype within the lesions. Another study focused on the phenotypic characterization of slowly expanding/smoldering lesions in PMS patients (71). Here, histological analysis also revealed that these lesions mainly composed pro‐inflammatory TMEM119^+^CD163^−^CD206^−^ microglia. Furthermore, as mentioned above, in active demyelinated lesions of human MS samples, CCL4^+^ TMEM119^+^ clusters of microglia are found, translating experimental mouse data into a human setting (58). Others have concentrated on the phenotype of peripheral myeloid cells, since these are much easier to access than brain tissue. Sc‐RNA‐Seq of myeloid cell in the cerebrospinal fluid (CSF) of MS patients characterized distinct subsets, with a monocyte cluster expressing the CAM markers LYVE1 and STAB1 as well as microglia markers TREM2 and TMEM119. A novel large‐scale CYTOF study reported a GM‐CSF producing CD4^+^ T cell subset expressing CXCR4 in MS patients (38). Ligands for CXCR4 are enriched in CSF and CNS tissue of MS patients and might be a gateway for these cytotoxic T cells into the brain (95). Interestingly, several studies have highlighted the importance of the meninges and potentially of CAM during MS (66, 92, 97, 112, 203). However, to our knowledge no data are available on the importance or phenotype of CAM in human MS. Taken together, the newly established and feasible methods have immensely increased our knowledge of myeloid cell phenotypes in MS patients. In combination with the data acquired from rodent models, we are coming closer to finding medical treatments options.

## Myeloid Cells as Treatment Targets

Most drugs, which are currently available, are DMF to treat RRMS. The progressive form of MS is still not well understood and RRMS DMF treatment has limited therapeutic benefits (182). Most of the current DMF do not target myeloid cells specifically but some can impact their activity. Different aspects of therapeutic myeloid cell targeting during CNS diseases have been reviewed in several publications in the past but this topic is exciting and highly dynamic (11).

Interferon‐β (IFN‐β) treatment is still the standard of care, although it also induces severe side effects (182). IFN‐β signaling on myeloid cells is critical for EAE development/progression (157). Type I IFNs are recognized ubiquitously by every cell, which can induce severe side effects if used systemically. The latest research has focused on the development of a cell type‐specific treatment option with IFN. Low‐affinity IFN is coupled to a cell type‐specific antibody, and therefore, elicits its function only upon binding to a specific surface receptor of a cell (25). Consequently, side effects are diminished, with positive outcomes seen in instances with CLEC9A‐specific binding to DCs which reduces EAE severity. Authorized DMF, such as Natalizumab and Cladribine mainly target lymphocytes, but have microglia‐modifying properties (5, 60, 75, 116, 177). The same holds true for the antibiotic minocycline and the DMF Laquinimod (81, 90, 124, 132, 176). However, in the latest clinical trials, these drugs have failed to improve disease progression, showing no convincing evidence to be used in MS treatment regiments (32). The sphingosine‐1‐phosphate receptor agonist Fingolimod and its more specific second generation inhibitors Ozanimod, Ponesimod, Amiselimod, and Siponimod, not only stop T cell trafficking to the CNS, but also lead to a decreased pro‐inflammatory response in microglia, which is neuroprotective (30, 78). Importantly, these drugs are also approved for SPMS (32).

Myeloid cells have been shown to play an important role in MS/EAE as described above and should, therefore, be exploited for future drug applications. It might be beneficial to stop peripheral myeloid cells from CNS entry. Additionally, it might help support local microglia/CAM in the CNS to stop inflammation and demyelination. Cell type‐specific targeting, however, might be a difficult endeavor in this scenario. For instance, ultrasmall iron particles are taken up by microglia as well as neutrophils and macrophages in EAE, indicating that targeting of myeloid cells in the CNS is possible (32, 88). Furthermore, the CSF1R kinase inhibitor BLZ945 induces brain region‐specific enhancement of remyelination and prevention of demyelination by depleting myeloid cells (7). A P2X4R agonist (Ivermectin, IVM), which is a FDA‐approved anti‐parasitic agent, ameliorated EAE, and LPC‐induced demyelination (201). One has to keep in mind, that in this study, myeloid cells were not differentiated into peripheral and resident cells for mechanistic analysis. Dimethyl fumarate and its second generation variant dimoxirel fumarate downregulates glycolysis in myeloid and lymphoid cells, thereby eliciting anti‐inflammatory and cytoprotective functions (33, 93). DMF treatment increases ROS production in peripheral monocyte, which coincided with decreased lymphocyte numbers in the blood of MS patients and outcome prediction (24). Because of the design of this human study (repetitive sampling over time), it focused on myeloid cells in the periphery, whereas another recent study has also focused on the role of oxidative stress of myeloid cells in neuroinflammation, but in a mouse model (121). This study indicated that myeloid cells within the CNS induce oxidative stress and disease progression, which can be ameliorated in several disease models by blocking antioxidant glutathione degradation using acivicin (121). After LPC‐dependent demyelination, niacin (vitamin B3) treatment, which is a clinically approved medication, inducing scavenger receptor CD36 expression, rejuvenated moMΦ/microglia, and enhanced remyelination via increased phagocytosis (158). Furthermore, upon demyelination microglia undergo necroptosis before the CNS is repopulated by anti‐inflammatory local microglia proliferation (105). Finally, neural stem cell therapies have also been proposed for P‐MS treatment (150) and induced a metabolic shift in moMΦ/microglia toward an anti‐inflammatory phenotype (MRC1^+^, iNOS^low^). This leads to ameliorated EAE severity, but most probably, was a complementary effect of direct action of NSC and the anti‐inflammatory phenotype of myeloid cells (148). Overall, immense progress has been made in the development and understanding of DMF, which target myeloid cell functions. Nevertheless, until now, no drugs are available which specifically target myeloid cell function.

## Conclusion

CNS‐resident myeloid cells as well as peripheral myeloid cells have been shown to be of utmost importance during disease progression of MS as well as in its rodent models. The evolution of single‐cell high‐throughput technologies/platforms, such as CYTOF, imaging mass cytometry, and sc‐RNA‐Seq has allowed the dissection of the myeloid compartment of the CNS in greater detail. It is clear now, that the landscape of the CNS is highly heterogeneous and that disease‐specific subsets of myeloid cells, resident as well as peripheral, can be distinguished and they show important diverse phenotypes and functions. It is becoming clearer that distinct functions are attributed with spatial location of these specific subsets. Importantly, unbiased sampling, especially in single‐cell analysis, often leads to under‐sampling of specific cell types and might misdirect our mechanistic understanding of the disease. Most importantly, the next step must be the functional analysis of these subsets in order to give us an idea about medical targeting. Targeting of specific myeloid cell subsets at certain time points during disease progression or in specific locations of pathologic events, might allow us to either stop their detrimental function or to push them toward a protective as well as resolving phenotype at the right time of disease state. In order to analyze the function of these cells, new targeting strategies will have to be developed to differentiate these time‐dependent subsets during the disease course.

## Summary Points

1. During homeostasis, myeloid cell subsets express specific core gene signatures
2. Upon inflammation and resolution, homeostatic core genes are downregulated and disease‐associated genes upregulated, making the subsets difficult to dissect
3. Newly generated sophisticated mouse models will make it possible to dissect distinct myeloid cell subset functions during inflammation

## Conflict of Interest

The authors have nothing to disclose.
